# Incidence trends of type 1 diabetes before and after the reunification in children up to 14 years of age in Saxony, Eastern Germany

**DOI:** 10.1371/journal.pone.0183665

**Published:** 2017-09-07

**Authors:** Ulf Manuwald, Peter Heinke, Eckhard Salzsieder, Janice Hegewald, Olaf Schoffer, Joachim Kugler, Thomas M. Kapellen, Wieland Kiess, Ulrike Rothe

**Affiliations:** 1 Health Sciences, Public Health, Faculty of Medicine “Carl Gustav Carus”, TU Dresden, Dresden, Germany; 2 Institute of Diabetes “Gerhardt Katsch” Karlsburg, Karlsburg, Germany; 3 Institute and Policlinic for Occupational and Social Medicine, Faculty of Medicine “Carl Gustav Carus”, TU Dresden, Dresden, Germany; 4 Center of Evidence-Based Healthcare, University Hospital “Carl Gustav Carus”, TU Dresden, Dresden, Germany; 5 Hospital for Children and Adolescents, Center for Pediatric Research, Department of Women and Child Health, University of Leipzig, Leipzig, Germany; German Diabetes Center, Leibniz Center for Diabetes Research at Heinrich Heine University Düsseldorf, GERMANY

## Abstract

**Aims:**

The aim of this study was to analyze the incidence rates of type 1 diabetes in Saxony before and after the German reunification.

**Methods:**

The study examined two registries: one until 1990 and one since 1999. Only patients under 15 years of age with type 1 diabetes and living in Saxony were included in the study. Standardized incidence rates were described based on direct age standardization procedures using the Standard European Population for each calendar year between the observation periods 1982–1989 and 1999–2014. Age was grouped into three classes: 0–4, 5–9 and 10–14 years of age. Incidence data were presented as age-standardized incidence rates per 100,000 person-years (PY) with 95% confidence intervals [CI]. Joinpoint regression was used for trend analyses and Poisson regression was used to adjust for the effects of age and sex on the incidence.

**Results:**

A total number of 2,092 incident cases of type 1 diabetes (1,109 males; 983 females) were included. The age-standardized incidence rates of type 1 diabetes per 100,000 PY was 7.9 [95%CI 6.8; 8.9] in the period from 1982–1989 and 20.1 [95%CI 14.0; 26.1] in the period from 1999–2014. The yearly increase in incidence over the entire time period (1982–2014) was 4.3% according to the average annual percent change (AAPC) method, and estimated to be 4.4% [95% CI 4.0; 4.8%] using a Poisson regression model adjusting for sex and age group.

**Conclusion:**

In this study, a significantly increasing incidence of type 1 diabetes was observed after reunification. In future studies it would be interesting to follow up on the question of which environmental and lifestyle factors could be causing the increasing type 1 diabetes incidence.

## Introduction

The incidence rates of type 1 diabetes vary strongly in different regions of the world. The highest incidence rates of type 1 diabetes are found in northern Europe (36.5/100,000 per year in Finland) and the lowest incidence rates are in China (0.1/100,000 per year) and South America (Venezuela 0.1/100,000 per year) [1, 2]. This north-south-gradient of disease distribution is well known [3]. Interestingly, in Europe and even within Germany, a west-east-gradient has also been detected, with lower incidence rates consistently observed in the east [3, 4]. Additionally, the annual incidence rates of type 1 diabetes in Europe have been increasing in children over time (time period 1989–2003; ranging from 0.6% to 9.3%), especially in the eastern countries (9.3% in Poland in the time period 1994–1998) [3–7].

The causes for the increasing incidence rates and the varying geographical distribution of incidence are still unknown. Different factors contributing to the increase of type 1 diabetes are discussed in the literature [8–14]. Environmental factors [8] such as ozone [9] and ultraviolet B irradiance and vitamin D levels in pregnant women [10] are discussed as possible contributing factors, as well as genetic factors [11, 15], the intestinal microbiome [12], parental age and increasing birth weight [13]. On the other hand, breast-feeding appears to reduce the risk of type 1 diabetes [14].

Following the German reunification in 1990, the environmental factors and living conditions in Eastern Germany changed considerably within a short period of time. These extraordinary circumstances provide an interesting opportunity to examine the impact of changing environmental exposures and social conditions on the incidence of type 1 diabetes. Prior to the reunification, the Central Diabetes-Registry Karlsburg recorded type 1 diabetes incidence from 1982 to 1989 in all children under the age of 15 years living in the German Democratic Republic (GDR). Since 1999, type 1 diabetes incidence in Saxony is monitored by the Childhood Diabetes Registry of Saxony. The aim of this study was to analyze the incidence rates of type 1 diabetes in Saxony before and after the German reunification by comparing the pediatric registry data.

## Materials and methods

### Data sources

Until the end of 1989, patient characteristics and the health status of all persons with diabetes from all 227 districts of the GDR were reported and recorded annually in the Central Diabetes-Registry Karlsburg. The districts Leipzig, Dresden and Chemnitz (previously Karl-Marx-Stadt) composed the present federal state of Saxony. The prevalence, incidence and the number of deaths of all persons with diabetes were registered. The registered persons with diabetes were stratified by age, sex and strategy of treatment. The Central Diabetes-Registry Karlsburg had a completeness of ascertainment of approximately 98% [16; 17]. Unfortunately, no external data sources were available to permit verification of the Central Diabetes Registry Karlsburg completeness of ascertainment. Instead, the ascertainment of each year was assessed by comparing the number diabetes patients registered in a certain year with the number expected from the preceding year’s data after accounting for deaths, remissive cases, and newly identified cases. According to this method, on average 0.98% (range: 0.03%-2.74%) of the expected cases were not identified [17]. The definition of type 1 diabetes used until 1989 was the presence of a permanent need for insulin therapy in children.

Post-reunification data were available from the Childhood Diabetes Registry of Saxony, which provides valid data for children aged 0–14 years since 1999. In Saxony, all children with diabetes are referred to a pediatric diabetologist working at one of 31 pediatric hospitals, and all of the pediatric hospitals regularly report new cases of diabetes to the registry where the data are checked for plausibility. Each year, all reported patients are verified with an inquiry (smaller hospitals) or checked against the cases registered in the *Diabetes-Patienten-Verlaufsdokumentation* (DPV, English: Diabetes Patient Clinical Documentation) database (larger hospitals) for completeness. The date of birth, sex, birth weight, residential postcode, family history of type 1 diabetes, date of manifestation, was well as clinical and biochemical data at the onset of diabetes were recorded using a standardized form. The Childhood Diabetes Registry of Saxony had a completeness of ascertainment of approximately 94% and 97% [4; 18] for children with diabetes under the age of 15 years (following the capture-mark-recapture method). Since the 1990s, type 1 diabetes was defined according to the EURODIAB criteria [19].

### Population at risk

Pre-unification population data were obtained from the statistical yearbook of the GDR and post-reunification from the Statistical State Office of Saxony. In Saxony, the population under the age of 15 years decreased from 934,929 in 1982 to 436,305 in 2005. Since 2005, the population under the age of 15 years in Saxony has been slowly increasing to 504,802 in 2014.

### Statistical analysis

Incidence rates were described based on direct age standardization procedures [20] for each calendar year between the observation periods 1982–1989 and 1999–2014. Age was grouped into three classes, 0–4, 5–9 and 10–14 years of age. All incidence rates are age-standardized using the Standard New European Population (www.gbe-bund.de). Incidence data were presented as age-standardized incidence rates per 100,000 person-years (PY) with 95% confidence intervals [CI] estimated using the normal approximation. Pooled incidence rates were compared between the periods 1982–1989 and 1999–2014.These calculations were performed with the spreadsheet program Excel 2010.

Trend analyses for incidence rates were performed using joinpoint regression, which is broadly used in cancer epidemiology. Average annual percent change (AAPC) and the respective 95% confidence intervals [CI] were estimated for the complete observations between 1982–2014 as well as the distinct observation periods 1982–1989 and 1999–2014. The fitted trend function is ln(y) = mx+b. Based on the slope parameter m the annual percent change (APC) is the transformation (exp(m)-1)*100. Whether the trend changes over time was investigated for the complete time period. The AAPC is the average of APCs for distinct time periods with different trends. These calculations were performed with the Joinpoint Regression Program (Version 4.2.0.2, Statistical Research and Applications Branch, National Cancer Institute, Bethesda, Maryland, USA).

The effects of age, sex and calendar periods on the incidence were modelled using Poisson regression with the population size as offset. Therefore, the estimates of incidence were adjusted for sex and age categories. The Poisson regression was performed using SAS (release 9.2).

### Ethics statement

This article does not contain any studies with human or animal subjects performed by any of the authors. The Childhood Diabetes Registry of Saxony was approved by the Ethical Committee of the Medical Faculty of the University of Leipzig (Reg. Nr. 981) and written informed consent was obtained from all parents.

## Results

In our study, a total number of 2,091 incident cases of type 1 diabetes (1,109 males; 982 females) were included; before reunification (1982–1989) 274 males and 285 females and after reunification (1999–2014) 835 males and 697 females.

**Fig 1:**
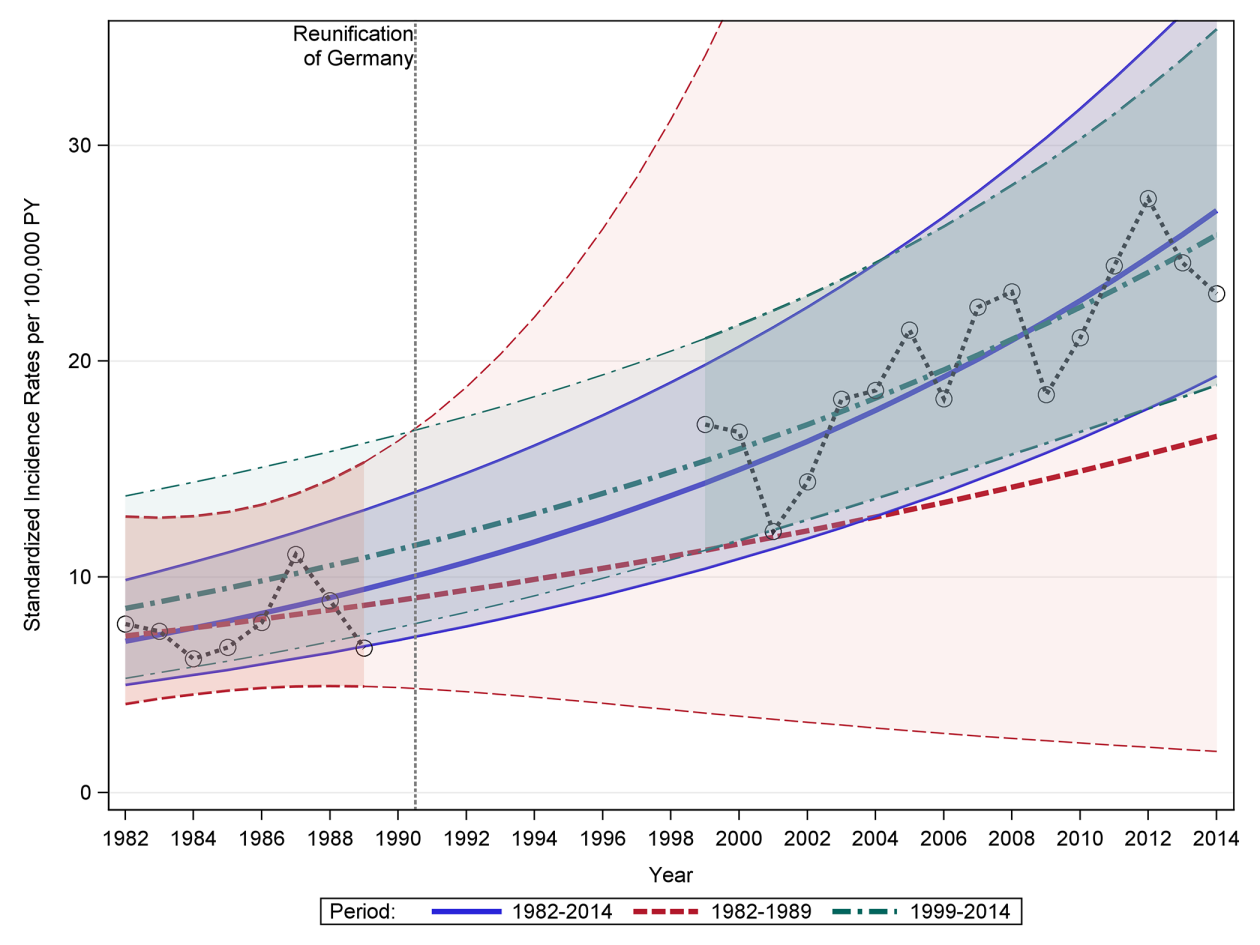
Modelled standardized incidence rates of type 1 diabetes in Saxony among children under the age of 15 years before and after reunification based on joinpoint regression. Purple solid lines: modelled standardized incidence rates and 95% CI based on all data (1982–2014); red dashed lines: modelled standardized incidence rates and 95% CI based only on data from 1982–1989; green dashed lines: standardized incidence rates and 95% CI based only on data from 1999–2014

The yearly number of cases, population at risk and standardized incidence rates are presented in (S1 Table) and Fig 1.

The age-standardized incidence rates of type 1 diabetes increased within and between the periods for children less than 15 years of age both before and after the reunification, but at different rates. The age-standardized incidence rates of type 1 diabetes per 100,000 PY was 7.9 [95% CI 6.8; 8.9] in the period from 1982 to 1989, and 20.1 [95% CI 14.0; 26.1] in the period from 1999 to 2014 (Table 1).

**Table 1:** Age-standardized incidence rates of type 1 diabetes among children under 15 years of age per 100,000 PY by sex, age groups and time period.

	Period	Age-standardized incidence rate per 100,000 PY [95%CI]	Cases	Population at risk
**Sex**				
Male	1982–1989	7.5 [4.9; 10.2]	274	3,767,121
	1999–2014	21.5 [12.7; 30.2]	835	3,971,603
	**1982–2014**	**16.8 [7.6; 26.0]**	**1109**	**7,738,724**
Female	1982–1989	8.2 [5.4; 11.0]	285	3,582,483
	1999–2014	18.6 [10.2; 27.0]	697	3,778,942
	**1982–2014**	**15.2 [6.3; 24.0]**	**982**	**7,361,425**
**Age group**				
0–4 years	1982–1989	3.8 [3.1; 4.6]	99	2,572,789
	1999–2014	14.0 [11.2; 16.9]	368	2,611,958
	**1982–2014**	**10.6 [7.7; 13.6]**	**467**	**5,184,747**
5–9 years	1982–1989	7.9 [6.8; 9.0]	194	2,446,444
	1999–2014	21.9 [18.1; 25.6]	532	2,394,378
	**1982–2014**	**17.2 [13.3; 21.1]**	**726**	**4,840,822**
10–14 years	1982–1989	11.5 [10.1; 12.9]	266	2,330,371
	1999–2014	24.0 [20.1; 27.8]	632	2,751,668
	**1982–2014**	**19.8 [15.7; 23.9]**	**898**	**5,082,039**
**Total** **(0–14 years)**	1982–1989	7.9 [5.9; 9.8]	559	7,349,604
	1999–2014	20.1 [14.0; 26.1]	1532	7,758,004
	**1982–2014**	**16.0 [9.6; 22.4]**	**2091**	**15,107,608**

In all age groups the standardized incidence rates of type 1 diabetes increased since the first period before the reunification of Germany. In the period 1982–1989 the incidence rates ranged from 3.5 to 11.5, and in the period 1999–2014 they ranged from 14.0 to 24.0. A differential examination of the incidence according to sex found incidence rates over both time periods (1982–2014) of 16.8 [95% CI 7.6; 26.0] and 15.2 [95% CI 6.3; 24.0] for male and female children, respectively.

In the total period from 1982 to 2014 (children under 15 years) the AAPC is 4.3% (Fig 1; Table 2), and no change in trend could be detected over this time period. The AAPC calculated based on the data from the GDR register was 2.6%, and the AAPC based on data from after 1999 was 3.5%. Considering only the years 1999–2014, a larger increase in incidence was detected for the age group of children aged 10–14 years than for the other age groups (i.e. ages 0–4 and 5–9 years). However, the differences in trend observed between sexes, time periods, and the age groups cannot be considered statistically significant (the confidence intervals are overlapping).

**Table 2:** Results of the trend analysis with joinpoint regression by sex, age groups and time period.

	Period	AAPC [95%CI]
**Sex**		
Male	1982–1989	-1.5 [-8.1; 5.6]
	1999–2014	3.4 [1.9; 4.9]
	**1982–2014**	**4.6 [3.9; 5.8]**
Female	1982–1989	7.0 [-1.9; 16.6]
	1999–2014	3.7 [1.6; 5.8]
	**1982–2014**	**3.9 [3.1; 4.7]**
**Age group**		
0–4 years	1982–1989	2.5 [-16.9; 26.4]
	1999–2014	1.7 [-0.8; 4.3]
	**1982–2014**	**4.9 [3.5; 6.4]**
5–9 years	1982–1989	-1.0 [-9.2; 8.0]
	1999–2014	3.5 [1.7; 5.3]
	**1982–2014**	**4.6 [3.8; 5.3]**
10–14 years	1982–1989	5.6 [0.0; 11.6]
	1999–2014	4.5 [2.5; 6.6]
	**1982–2014**	**3.7 [3.0; 4.4]**
**Total** **(0–14 years)**	1982–1989	2.6 [-4.9; 10.7]
	1999–2014	3.5 [2.1; 5.0]
	**1982–2014**	**4.3 [3.7; 4.9]**

The results of the Poisson regression models are shown in Table 3. The yearly increase in incidence adjusting for sex and age group over the entire time period (1982–2014) was 4.4% [95% CI 4.0; 4.8%]. A stratified examination of the two time periods with available data show differences in the yearly increase, as well as the sex- and age-associated risks. The yearly increase was estimated to be 2.3% [95% CI -1.3; 6.0] in the years 1982–1989 and 3.6% [95% CI 2.5; 4.7] in the years 1999–2014. The incidence rates among chidren aged 0–4 years were consistently and statistically significantly lower than those of both other age groups (5–9 and 10–14 years) for the entire time period (1982–2014) and when both time periods are examined seperately. There was no statistically significant difference in incidence rates between male and female children over the years 1982–2014 (male vs. female (reference): 1.075 [95% CI 0.986; 1.171].

**Table 3:** Results of Poisson regression the entire time period (1982–2014) and stratified according to the time periods with data (1982–1989 and 1999–2014).

	Rate ratio [95% CI]
**Years 1982–1989**	
Year (continuous)	1.023 [0.987; 1.060]
Male	0.914 [0.774; 1.079]
Female	Reference
Age group 10–14 years	2.971 [2.358; 3.742]
Age group 5–9 years	2.050 [1.060; 2.612]
Age group 0–4 years	Reference
**Years 1999–2014**	
Year (continuous)	1.036 [1.025; 1.047]
Male	1.140 [1.031; 1.261]
Female	Reference
Age group 10–14 years	1.697 [1.491; 1.930]
Age group 5–9 years	1.571 [1.375; 1.794]
Age group 0–4 years	Reference
**Total:**	
**Years 1982–2014**	
Year (continuous)	1.044 [1.040; 1.048]
Male	1.075 [0.986; 1.171]
Female	Reference
Age group 10–14 years	1.971 [1.762; 2.204]
Age group 5–9 years	1.668 [1.485; 1.874]
Age group 0–4 years	Reference

## Discussion

In Saxony, we observed a significant rise of type 1 diabetes incidence since 1999 until 2014 compared to the time before the reunification of Germany. Before Germany’s reunification, Saxony was a part of the Eastern Germany (GDR) and the age-standardized incidence rates of type 1 diabetes was low; now, after the reunification, the age-standardized incidence rates are three times higher than before. Our observations are consistent with the higher annual rising of age-standardized incidence rates in Eastern European countries described by Patterson et al. [3].

According to the trend analysis, a positive trend over time can be observed, but no change in the trend over time was detected. However, due to the overlapping confidence intervals, the differences in trends observed between sexes, time periods, and the age groups cannot be considered statistically significant. Moreover, due to the lack of data no statement can be made about the exact development of trends between 1989 and 1999. It is unclear whether the course modelled by joinpoint regression and depicted in Fig 1 is accurate or if an abrupt increase may have occurred during this time period. Thus, only speculations about the general trend of the incidence rates are possible. In particular two scenarios seem conceivable. On the one hand, the trend of the incidence rates during the GDR could be completely different from the trend of the incidence rates after reunification, resulting in a change in the trend (structural change). On the other hand, the course could correspond to a continuous percentage increase over the entire time. Then there would be no change in the trend between the GDR and the time after reunification.

The age-standardized incidence rates of type 1 diabetes differed in the three age groups (0–4, 5–9, 10–14 years); the higher the age group the higher the incidence rate. These results reflect similar observations found in Austria [21]. In our analysis the highest increase of age-standardized incidence rates was in the group of 10–14 years, which differed significantly to from the age group of 0–4 years. The presence of an age-related difference in incidence rates was also confirmed by the results of the Poisson regression where the older age groups had higher estimated incidence rate ratios compared to the age group of 0–4 years. This finding is in contrast to the results of Ehehalt et al. 2010 [22]. They predicted the greatest increase of age-standardized incidence rate would be observed among children aged 5–9 years in the years 2006–2010 based on the observed incidence before 2006 for Baden Wuerttemberg, Germany.

The availability of incidence data for Saxony before and after the German reunification offered a unique opportunity to observe differences in disease incidence before and after a substantial political restructuring with wide-randing societal and economic repercussions. Major changes in living and environmental conditions occurring since the reunification may have influenced the significant increase in incidence [18, 23]. Additionally, the average age of women at the time of the first pregnancy has been increasing rapidly in Eastern Germany since the reunification. Before 1990, the average age of first time mothers was 22.9 years, and after the reunification the average age increased to 26.9 years by 1995 [24]. An increasing average parental age at the time of the first pregnancy [21, 25] and higher birth weights [13, 26] are also discussed in the literature as possible hypotheses for the increase of type 1 diabetes. In Austria the average age of mothers at first pregnancy has also increased from 24.0 years in 1985 to 26.2 years in 1995 [27]. Likewise during the 1990s a significant increase of type 1 diabetes incidence in Austria and France was observed [21, 28]. Patterson [3] and Bendas et al. [4] detected a west-east gradient of SIR for type 1 diabetes that has been decreasing over time (after the 1990s and after 2000, respectively). These results are in line with our results and suggest that the observed regional differences may have been due to divergent social and environmental situations.

Similar patterns were also observed with other auto-immune diseases, such as the increase in allergies in children observed after the reunification [29; 30].

### Strengths and limitation

#### Strengths of our study

The study is based on data from population-based diabetes registries with excellent completeness of ascertainment and data quality. The pre-unification registry provides valuable information regarding type 1 diabetes morbidity prior to the German reunification, and altogether the registry data depict 24 years of disease incidence.

#### Limitations

Unfortunately, one major limitation is the lack of registry data for the years between 1989 and 1999 for Saxony. Thus, we cannot describe the disease incidence development in detail in this 9 year period. Another limitation is the lack of data regarding environmental exposures and lifestyle factors in both registries. Thus, the possible reasons for the increasing incidence trends of type 1 diabetes in Saxony cannot be explained at this time. An additional factor that could have also affected the observed age-standardized incidence rate is the migration from west to east (and east to west) that was first largely possible after the reunification.

Due to the statistical noise associated with a low number of cases, especially for the period before the reunion, artifacts of the development over time cannot be ruled out. Therefore, trend statements regarding the GDR must be interpreted with caution.

Another minor limitation is the lack of yearly ascertainment of registry completeness and information regarding completeness in the different age groups. However, the available ascertainment completeness assessments indicate that the registry completeness is good, and we have no reason to believe that fluctuations in ascertainment completeness could have greatly impacted the observed incidence rates.

## Conclusions

In conclusion, a significantly increasing incidence of type 1 diabetes was observed after reunification. Future studies are needed to examine the etiological role of environmental and lifestyle factors on type 1 diabetes incidence in order to find ways to effectively prevent the onset of the disease.

## Supporting information

S1 Table (DOCX)

## References

[1] Karvonen M, Viik-Kajander M, Moltchanova E, Libman I, LaPorte R, Tuomilehto J. Incidence of childhood type 1 diabetes worldwide. Diabetes Mondiale (DiaMond) Project Group. Diabetes Care. 2000;23(10):1516–26. 11023146 10.2337/diacare.23.10.1516

[2] Soltesz G, Patterson CC, Dahlquist G Worldwide childhood type 1 diabetes incidence- what can we learn from epidemiology?. Pediatr Diabetes 8 2007;Suppl 6:6–14. 17727380 10.1111/j.1399-5448.2007.00280.x

[3] Patterson CC, Dahlquist GG, Gyurus E, Green A, Soltesz G. Incidence trends for childhood type 1 diabetes in Europe during 1989–2003 and predicted new cases 2005–20: a multicentre prospective registration study. Lancet. 2009;373(9680):2027–33. doi: 10.1016/S0140-6736(09)60568-7 19481249 10.1016/S0140-6736(09)60568-7

[4] Bendas A, Rothe U, Kiess W, Kapellen T M, Stange T, Manuwald U et al Trends in Incidence Rates during 1999–2008 and Prevalence in 2008 of Childhood Type 1 Diabetes Mellitus in Germany—Model-Based National Estimates. PloS one. 2015;10(7):e0132716 doi: 10.1371/journal.pone.0132716 26181330 10.1371/journal.pone.0132716 PMC4504467

[5] Patterson CC, Gyurus E, Rosenbauer J, Cinek O, Neu A, Schober E, et al Trends in childhood type 1 diabetes incidence in Europe during 1989–2008: evidence of non-uniformity over time in rates of increase. Diabetologia. 2012;55(8):2142–7. doi: 10.1007/s00125-012-2571-8 22638547 10.1007/s00125-012-2571-8

[6] EURODIAB ACE Study Group. Variation and trends in incidence of childhood diabetes in Europe. EURODIAB ACE Study Group. Lancet. 2000;355(9207):873–6. 10752702

[7] Green A, Patterson CC, Europe ETSG. Diabetes trends in the incidence of childhood onset diabetes in Europe 1989–1998. Diabetologia. 2001;44 Suppl 3:B3–8. 11724413 10.1007/pl00002950

[8] Bodin J, Stene LC, Nygaard UC. Can exposure to environmental chemicals increase the risk of diabetes type 1 development? Biomed Res Int. 2015;208947 doi: 10.1155/2015/208947 25883945 10.1155/2015/208947 PMC4391693

[9] Hathout EH, Beeson WL, Ischander M, Rao R, Mace JW. Air pollution and type 1 diabetes in children. Pediatr Diabetes. 2006;7(2):81–7. doi: 10.1111/j.1399-543X.2006.00150.x 16629713 10.1111/j.1399-543X.2006.00150.x

[10] Mohr SB, Garland CF, Gorham ED, Garland FC. The association between ultraviolet B irradiance, vitamin D status and incidence rates of type 1 diabetes in regions worldwide. Diabetologia. 2008;51(8):1391–8. doi: 10.1007/s00125-008-1061-5 18548227 10.1007/s00125-008-1061-5

[11] Hermann R, Knip M, Veijola R, Simell O, Laine AP, Akerblom HK et al Temporal changes in the 1 frequencies of HLA genotypes in patients with Type 1 diabetes—indication of an increased environmental pressure? Diabetologia. 2003;46(3):420–5. doi: 10.1007/s00125-003-1045-4 12687342 10.1007/s00125-003-1045-4

[12] Gulden E, Wong FS, Wen L. The gut microbiota and Type 1 Diabetes. Clin Immunol. 2015;5 159(2):143–53. doi: 10.1016/j.clim.2015.05.013 26051037 10.1016/j.clim.2015.05.013 PMC4761565

[13] Cardwell CR, Carson DJ and Patterson CC. Parental age at delivery, birth order, birth weight and gestational age are associated with the risk of childhood Type 1 diabetes: a UK regional retrospective cohort study. Diabet Med. 2005;22(2):200–6. doi: 10.1111/j.1464-5491.2005.01369.x 15660739 10.1111/j.1464-5491.2005.01369.x

[14] Cardwell CR, Stene LC, Ludvigsson J, Rosenbauer J, Cinek O, Svensson J et al Breast-feeding and childhood-onset type 1 diabetes: a pooled analysis of individual participant data from 43 observational studies. Diabetes Care. 2012;35(11):2215–25. doi: 10.2337/dc12-0438 22837371 10.2337/dc12-0438 PMC3476923

[15] Kukko M, Virtanen SM, Toivonen A, Simell S, Korhonen S, Ilonen J et al Geographical variation in risk HLA-DQB1 genotypes for type 1 diabetes and signs of beta-cell autoimmunity in a high-incidence country. Diabetes Care. 2004;27(3):676–81. 14988284 10.2337/diacare.27.3.676

[16] Michaelis D, Jutzi E, Heinke P. 30jähriger Inzidenz- und Prävalenztrend des juvenilen Typ-1-Diabetes in der ostdeuschen Bevölkerung. Diabetes und Stoffwechsel. 1993;2:245–250.

[17] Michaelis D, Jutzi E, Voigt L. Epidemiology of insulin-treated diabetes mellitus in the East-German population: differences in long-term trends between incidence and prevalence rates. Diabete & metabolisme. 1993;19(1 Pt 2):110–5. 8314412

[18] Galler A, Stange T, Müller G, Näke A, Vogel C, Kapellen T et al Incidence of childhood diabetes in 1 children aged less than 15 years and its clinical and metabolic characteristics at the time of diagnosis: data from the Childhood Diabetes Registry of Saxony, Germany. Horm Res Paediatr. 2010;74(4):285–91. doi: 10.1159/000303141 20516654 10.1159/000303141

[19] Green A, Gale EA, Patterson CC. Incidence of childhood-onset insulin-dependent diabetes mellitus: the EURODIAB ACE Study. Lancet. 1992;339(8798):905–9. 1348306 10.1016/0140-6736(92)90938-y

[20] Breslow NE, Day NE. Statistical methods in cancer research. Volume II—The design and analysis of cohort studies. IARC Sci Publ. 1987;(82):1–406. 3329634

[21] Schober E, Rami B, Waldhoer T. Steep increase of incidence of childhood diabetes since 1999 in Austria. Time trend analysis 1979–2005. A nationwide study. Eur J Pediatr. 2008;167(3):293–7. doi: 10.1007/s00431-007-0480-5 17453237 10.1007/s00431-007-0480-5

[22] Ehehalt S, Dietz K, Willasch AM, Neu A. Epidemiological perspectives on type 1 diabetes in childhood and adolescence in germany: 20 years of the Baden-wuerttemberg Diabetes Incidence Registry (DIARY). Diabetes Care. 2010;33(2):338–40. doi: 10.2337/dc09-1503 19903753 10.2337/dc09-1503 PMC2809277

[23] Ehehalt S, Neu A, Michaelis D, Heinke P, Willasch AM, Dietz K. Incidence of type 1 diabetes in childhood before and after the reunification of Germany—an analysis of epidemiological data, 1960–2006. Exp Clin Endocrinol Diabetes. 2012;120(8):441–4. doi: 10.1055/s-0032-1309045 22576256 10.1055/s-0032-1309045

[24] Pötzsch O. Geburten in Deutschland. Statistisches Bundesamt: Wiesbaden 2012;p.11.

[25] Bache I, Bock T, Volund A, Buschard K. Previous maternal abortion, longer gestation, and younger maternal age decrease the risk of type 1 diabetes among male offspring. Diabetes Care. 1999;22(7):1063–5. 10388968 10.2337/diacare.22.7.1063

[26] Stene LC, Magnus P, Lie RT, Sovik O, Joner G. Birth weight and childhood onset type 1 diabetes: population based cohort study. BMJ. 2001;322(7291):889–92. 11302899 10.1136/bmj.322.7291.889 PMC30582

[27] Kaindl M, Schipfer RK. Famielen in Zahlen 2014, A.I.f.F. Studies, Editor. Universität Wien, Wien 2014;p.18.

[28] Barat P, Valade A, Brosselin P, Alberti C, Maurice-Tison S, Levy-Marchal C. The growing incidence of type 1 diabetes in children: the 17-year French experience in Aquitaine. Diabetes Metab. 2008;34(6 Pt 1):601–5. 18952477 10.1016/j.diabet.2008.06.002

[29] Lehmann I, Thoelke A, Weiss M. T cell reactivity in neonates from an East and a West German city—results of the LISA study. Allergy. 2002;57(2):129–36. 11929415 10.1046/j.0105-4538.2002.00001.x

[30] Krämer U, Oppermann H, Ranft U, Schäfer T, Ring J, Behrendt H. Differences in allergy trends between East and West Germany and possible explanations. ClinExp Allergy. 2010 2;40(2):288–98. 10.1111/j.1365-2222.2009.03435.x 20210807

