# Supplementary material for: Incidence trends of type 1 diabetes before and after the reunification in children up to 14 years of age in Saxony, Eastern Germany
Source: PLoS One. 2017 Sep 7;12(9):e0183665. doi: 10.1371/journal.pone.0183665 (PMC5589116; doi:10.1371/journal.pone.0183665)
Supplement: S1 Table — (DOCX) [file pone.0183665.s001.docx]

**S1 Table: Age-standardized incidence rates of type 1 diabetes per 100,000 PY**

| **Age-standardized incidence rates** | **(95% CI)** | **Inhabitants under 15 years** | **Cases under 15 years** | **Year** |
| --- | --- | --- | --- | --- |
| 7.8 | (6.0-9.6) | 934,929 | 72 | 1982 |
| 7.5 | (5.7-9.2) | 931,396 | 68 | 1983 |
| 6.2 | (4.6-7.8) | 925,202 | 55 | 1984 |
| 6.7 | (5.0-8.4) | 919,095 | 60 | 1985 |
| 7.9 | (6.0-9.8) | 910,181 | 69 | 1986 |
| 11.1 | (8.8-13.3) | 908,154 | 97 | 1987 |
| 8.9 | (7.0-10.9) | 912,251 | 79 | 1988 |
| 6.7 | (5.0-8.5) | 908,396 | 59 | 1989 |
| … | … | … | … | … |
| 17.2 | (13.7-20.7) | 578,355 | 100 | 1999 |
| 16.7 | (13.2-20.2) | 550,835 | 97 | 2000 |
| 12.1 | (9.0-15.2) | 522,225 | 63 | 2001 |
| 14.4 | (11.0-17.8) | 494,070 | 72 | 2002 |
| 18.3 | (14.4-22.2) | 470,594 | 85 | 2003 |
| 18.6 | (14.6-22.6) | 451,952 | 83 | 2004 |
| 21.5 | (17.0-25.9) | 436,305 | 91 | 2005 |
| 18.2 | (14.1-22.3) | 437,421 | 77 | 2006 |
| 22.5 | (18.0-26.9) | 444,508 | 98 | 2007 |
| 23.2 | (18.7-27.7) | 454,198 | 103 | 2008 |
| 18.4 | (14.5-22.3) | 464,584 | 85 | 2009 |
| 21.0 | (16.8-25.1) | 476,168 | 99 | 2010 |
| 24.2 | (19.7-28.6) | 484,033 | 114 | 2011 |
| 27.3 | (22.7-32.0) | 491,926 | 132 | 2012 |
| 24.5 | (20.1-28.9) | 496,028 | 119 | 2013 |
| 23.1 | (18.9-27.4) | 504,802 | 114 | 2014 |
